# Supplementary material for: The Enhanced Thermoelectric and Mechanical Performance of Polythiophene/Single-Walled Carbon Nanotube Composites with Polar Ethylene Glycol Branched-Chain Modifications
Source: Polymers (Basel). 2024 Mar 29;16(7):943. doi: 10.3390/polym16070943 (PMC11013142; doi:10.3390/polym16070943)
Supplement: Supplementary file 1 [file polymers-16-00943-s001.zip › polymers-2608658-supplementary.pdf]

Supporting Information

The Enhanced Thermoelectric and Mechanical Performance of Polythiophene/Single-Walled Carbon Nanotube Composites with Polar Ethylene Glycol Branched-Chain Modifications

Qing Yang [1,†], Shihong Chen [1,†], Dagang Wang [1], Yongfu Qiu [2], Zhongming Chen [2], Haixin Yang [1], Xiaogang Chen [1], Zijian Yin [1] and Chengjun Pan [1,*]

[1] College of Chemistry and Chemical Engineering, Shenzhen University, Shenzhen 518060, China; 2110343048@email.szu.edu.cn (Q.Y.); chensher7@163.com (S.C.); wangdagang@szu.edu.cn (D.W.); 13720385426@163.com (H.Y.); 2110343112@email.szu.edu.cn (X.C.); 2110343060@email.szu.edu.cn (Z.Y.)

[2] School of Environment and Civil Engineering, Dongguan University of Technology, Guangdong 523808, China; qiuyf@dgut.edu.cn (Y.Q.); zmchen@dgut.edu.cn (Z.C.)

* Correspondence: pancj@szu.edu.cn

† These authors contributed equally to this work.

1. Materials and Synthesis

1.1. Materials

N-Bromosuccinimide (NBS) was purchased from Saen Chemical Technology (Shanghai) Co., Ltd. Chloro(1-methylethyl)magnesium (i-PrMgCl), Epichlorohydrin, Phosphorus tribromide (PBr_3), Triethylene glycol monomethyl ether, Sodium hydride (NaH), Potassium hydroxide (KOH), Sodium carbonate, Magnesium sulfate and Sodium thiosulfate were purchased from Anhui Zesheng Technology Co., Ltd. 3-Thiophenemethanol was purchased from Zhengzhou Alfa Chemical Co., Ltd. [1,3-Bis(diphenylphosphino)propane]nickel(II)chloride (Ni(dppp)Cl_2) was purchased from Tixiai (Shanghai) Chemical Industry Development Co., Ltd. All of the solvents were purchased from Anhui Zesheng Technology Co., Ltd, which were in anhydrous level.

1.2. Synthesis of 2,5-Dibromo-3-thiophenemethanol (1)

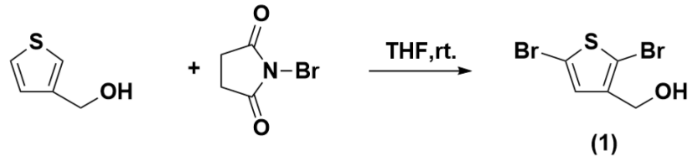

Scheme S1. Synthetic routes of **(1)**.

In a nitrogen-filled 250 mL flask, 10 g of 3-thienylmethanol (1 eq, 54.75 mmol) was dissolved in 50 mL of anhydrous tetrahydrofuran. Concurrently, 2.69 g of NBS (2.2 eq, 120.45 mmol) was suspended in 60 mL of anhydrous THF and incrementally added to the initial mixture at 0 °C, then allowed to stir overnight at room temperature. The reaction was extracted with hexane and quenched with a 10% sodium thiosulfate solution, then dried over anhydrous magnesium sulfate. After filtration and evaporation, the crude product was purified by column chromatography (ethyl acetate/hexane, 1:4), yielding 12.67 g (86%) of product **(1)** as a light yellow, transparent oil. ^{1}H NMR (500 MHz, CDCl3) δ 7.15 (s, 1H), 5.33 (t, J = 5.7 Hz, 1H), 4.33 (d, J = 5.5 Hz, 2H).

1.3. Synthesis of 2,5-Dibromo-3-(bromomethyl)thiophene (2)

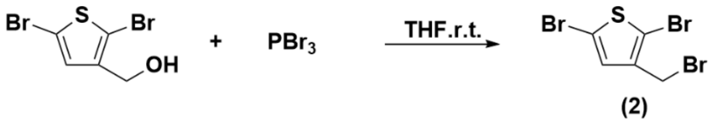

Scheme S2. Synthetic routes of **(2)**.

In a nitrogen-filled 250 mL flask, 10 g of **(1)** (1 eq, 36.75 mmol) was injected, followed by the addition of 150 mL of anhydrous dichloromethane. After **(1)** was completely dissolved, 10.95 g of PBr3(1.1 eq, 40.43 mmol) was gradually added in an ice-water bath. The reaction was allowed to proceed at room temperature in the dark for 5 hours after the ice bath was removed. The reaction was quenched with dichloromethane and a 10% sodium bicarbonate solution, followed by multiple washings with distilled water. The organic layer was dried over anhydrous magnesium sulfate, filtered, and the solvent was removed under reduced pressure. The crude product was purified by column chromatography (dichloromethane/hexane, 3:2) to yield 11.2 g (88%) of product **(2)** as a light yellow, transparent oil. ^{1}H NMR (500 MHz, CDCl3) δ 7.00 (s, 1H), 4.36 (s, 2H).

1.4. Synthesis of 2,5,8,11,15,18,21,24-Octaoxapentacosan-13-ol (3)

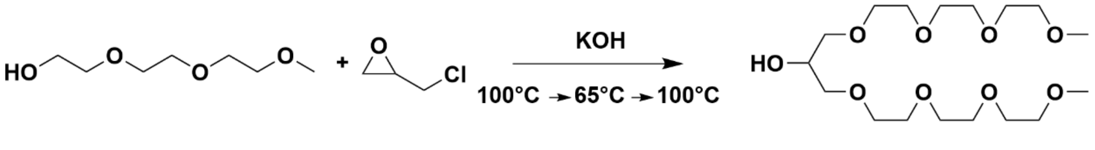

(3)

Scheme S3. Synthetic routes of **(3)**.

In a nitrogen-purged 250 mL flask, 30 g of trimethylolethane monomethyl ether (3.05 eq, 182.7 mmol) was heated to 100 °C and reacted with 3.53 g of KOH (1.05 eq, 62.98 mmol) for 3 hours. The mixture was cooled to 80 °C before the addition of 5.58 g of epichlorohydrin (1 eq, 60 mmol), then heated to 100 °C and stirred overnight. After cooling and quenching with methanol, the reaction was filtered, washed with dichloromethane, and rotary evaporated. The crude product was purified by distillation, yielding product **(3)** as a light yellow oil (12.63 g, 54.92%). ^1H NMR (500 MHz, $CDCl_3$) δ 3.99 (m, J = 6.4, 4.4, 2.0 Hz, 1H), 3.82-3.59 (m, 20H), 3.58-3.52 (m, 8H), 3.39 (s, 6H).

1.5. Synthesis of 3-(2-(2-(2-methoxyethoxy)ethoxy)ethoxy)methylthiophene (4) and 3-(1,3-bis(triethoxymethoxy)propan-2-yloxy)methylthiophene (5)

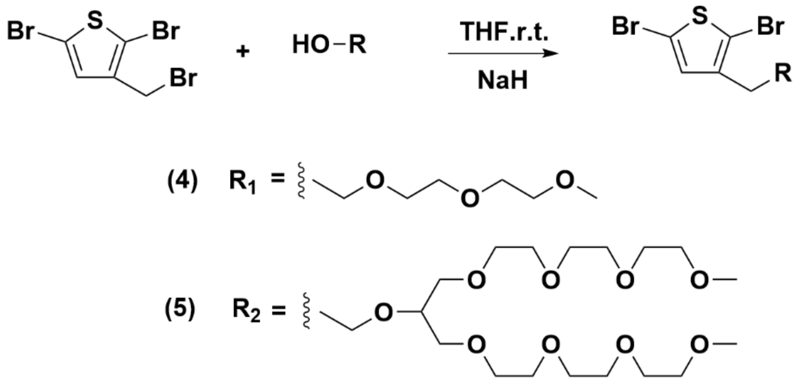

Scheme S4. Synthetic routes of **(4)** and **(5)**.

In a nitrogen-purged 50 mL flask K, 0.32 g of NaH (60% in oil, 1.5 eq, 8.07 mmol) was suspended in 3.5 mL of anhydrous THF. Simultaneously, in another dried 50 mL flask L, 1.8 g of trimethylolethane (1 eq, 5.38 mmol) was dissolved in 4.5 mL of anhydrous tetrahydrofuran after thrice purging with nitrogen. The solution from flask L was gradually added to flask K under an ice bath, stirring until bubbling ceased, then allowed to react for 60 minutes at room temperature. The intermediate, 1.05 g of **(2)** (1.2 eq, 6.456 mmol), was weighed in a 25 mL flask M, dissolved in 3.5 mL of anhydrous THF after triple nitrogen purging, and then slowly injected into flask K for overnight reaction at room temperature. After completion, the reaction mixture was filtered through diatomaceous earth, washed repeatedly with dichloromethane, then rotary evaporated. The crude product was purified via silica gel column chromatography, eluting with dichloromethane/ethyl acetate (3:2). The product **(4)** was obtained as a light yellow transparent oil (1.78 g, 84%). Product **(5)** was synthesized using the same procedure as product (4).^1H NMR (500 MHz, $CDCl_3$) of product **(4)** δ 6.99 (s, 1H), 4.43 (s, 2H), 3.66-3.60 (m, 10H), 3.56-3.53 (m, 2H), 3.37 (s, 3H). ^1H NMR (500 MHz, $CDCl_3$) of product **(5)** δ 7.05 (s, 1H), 4.56 (s, 2H), 3.76-3.69 (m, 1H), 3.67-3.54 (m, 28H), 3.37 (s, 6H).

1.6. Synthesis of P3(TEG)T and P3MBTEMT

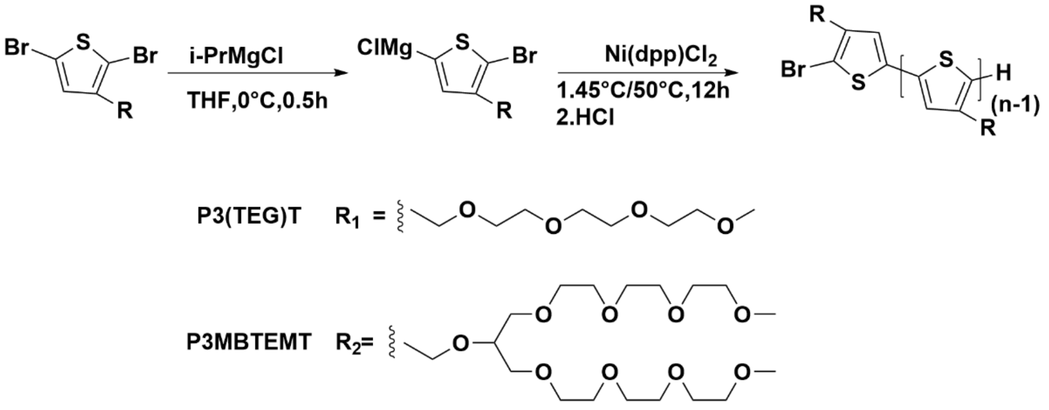

Scheme S5. Synthetic routes of P3(TEG)T and P3MBTEMT.

In a dry 50 mL flask, 520 mg of polymer **(4)** (1 eq, 1.29 mmol) was subjected to three vacuum-nitrogen cycles. 13 mL of anhydrous THF and 1.08 mL i-PrMgCl (0.98 eq, 1.26 mmol) were added under a nitrogen atmosphere and ice-bath conditions. After 30 minutes of dark reaction at room temperature, 6.97 mg of Ni(dppp)Cl_2 (0.0129 mmol) was introduced. The mixture was heated to 50 °C for 12 hours, quenched with 1 M HCl (0.5ml), and the product extracted until the aqueous phase was almost colorless. The oligomers were removed by repeated centrifugation (5800 rpm, 10 minutes), and dried under vacuum at 40 °C for 12 h, yielding a dark red polymer P3MBTEMT (250 mg, 75%). P3(TEG)T was synthesized using the same procedure as P3MBTEMT. ^{1}H NMR (500 MHz, $CDCl_3$) of P3(TEG)T δ 4.66 (s, 2H), 3.74-3.62 (m, 10H), 3.53-3.51 (m, 2H), 3.35 (s, 3H). ^{1}H NMR (500 MHz, $CDCl_3$) of P3MBTEMT δ 4.79 (s, 2H), 3.82 (m, 1H), 3.66-3.50 (m, 28H), 3.34 (s, 6H).

2. Characterisation and measurements

2.1. measurements

Characterization of synthesized small-molecule compounds and polymers was conducted using Nuclear Magnetic Resonance (NMR) spectroscopy on Bruker (500 MHz) with samples prepared as 5 mg mL^{-1} solutions in $CDCl_3$. Gel Permeation Chromatography (GPC) with Waters e2695 Separations Module was employed to study polymer molecular weight distribution, using samples prepared as 2 mg/mL solutions in chromatographically pure THF. Thermal stability of the samples was examined using a TGA-55 (American TA Instruments), with 10 mg of sample heated from 25 to 800 °C at a rate of 10 °C/min under nitrogen. UV-Vis Absorption Spectroscopy was studied using the Evolution 220 (U.S.A. Thermo Fisher Scientific Co., Ltd.) instrument. Samples were spin-coated onto substrates at 1800 r/min to obtain thin films for maximum absorption wavelength (λ_{abs}). Laser Raman spectroscopy was studied using a RENIDHAW in Via (Renishaw) with a 514 nm excitation source and measurement range of 1000-2000 cm^{-1}. Surface morphology was observed using a SU-70 scanning electron microscope (SEM) (Hitachi High-Technologies Corporation). X-ray diffraction (XRD) was performed on a SmartLab (Japan Rigaku Co., Ltd.), using a focusing beam and scanning from 5° to 60° at 10° min^{-1}. Tensile and bending tests were performed using a CMT4204 Universal Testing Machine (Shanghai Xinsansi Scientific Industrial Co., Ltd.). Tensile samples were prepared using PTFE molds into dumbbell-shaped, with 25 mm long and 4 mm wide narrow portions and 150 μm thickness. Thermoelectric performance (TE) of the thin film samples was measured using a MRS-3 (Wuhan Jiayitong Technology Co., Ltd.), and film thickness was measured using an ET-4000M two-dimensional micro-profile measuring instrument (Japan Kosaka Laboratory LTD). TE performance of the flexible TE devices was measured using a Keithley 4200A-SCS test platform (Tektronix Technology (China) Co., Ltd.).

2.2. Characterisation

2.2.1. Nuclear Magnetic Resonance (NMR)

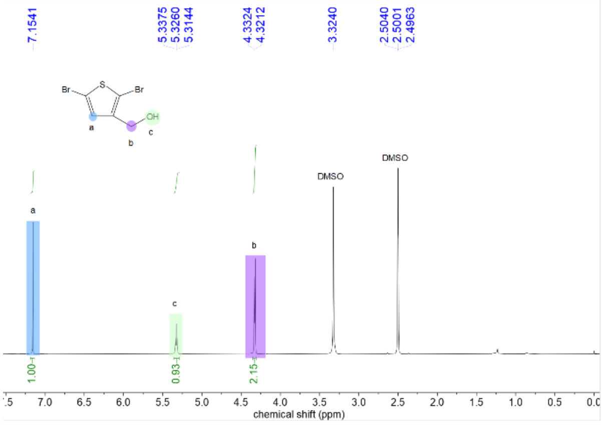

Figure S1. ^{1}H NMR of **(1)** in DMSO.

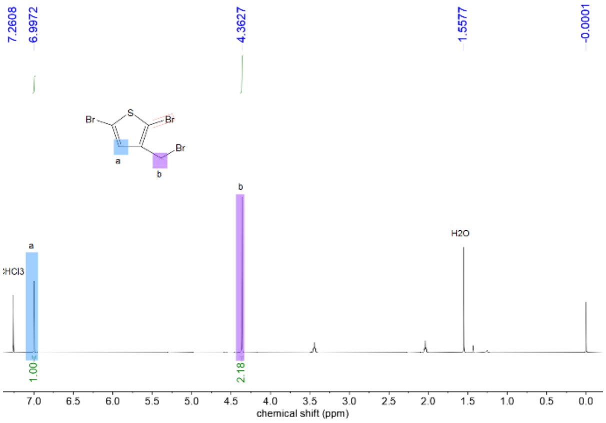

Figure S2. ^{1}H NMR of **(2)** in $CDCl_3$.

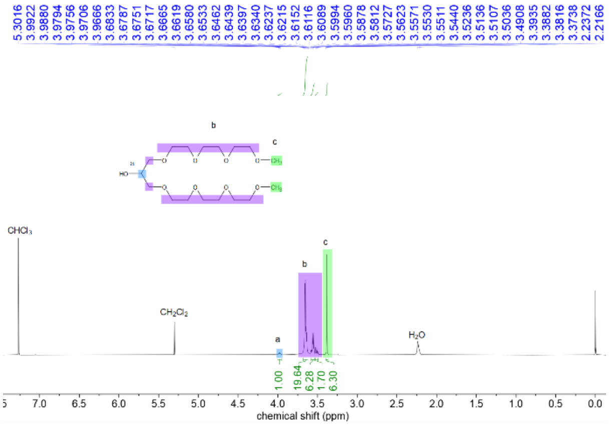

Figure S3. ^{1}H NMR of **(3)** in $CDCl_3$.

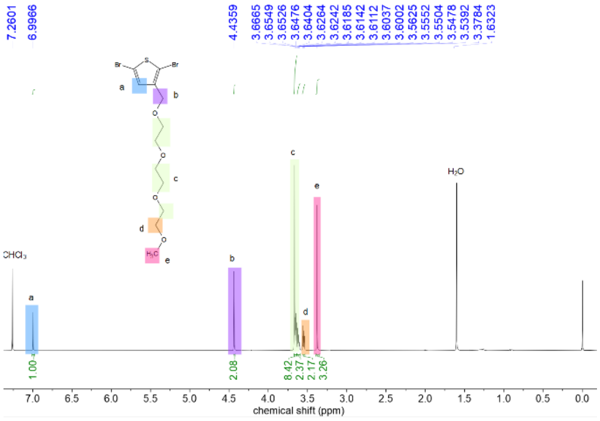

Figure S4. ^{1}H NMR of **(4)** in $CDCl_3$.

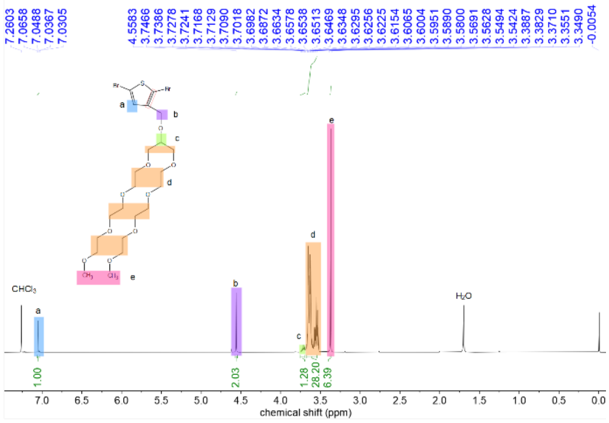

Figure S5. ^{1}H NMR of **(5)** in $CDCl_3$.

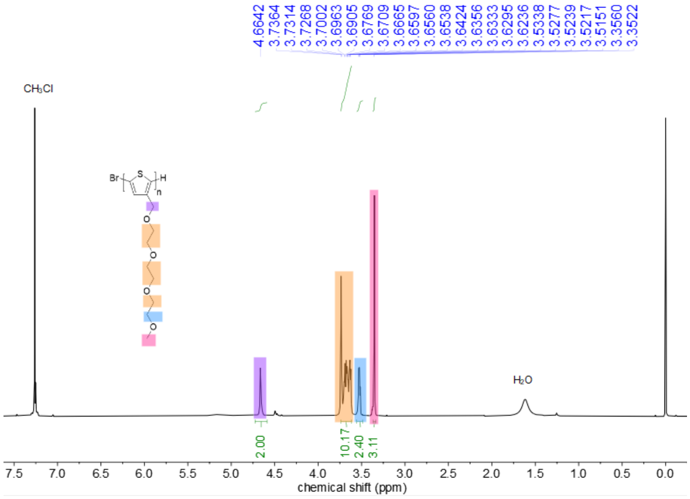

Figure S6. ^{1}H NMR of P3(TEG)T in $CDCl_3$.

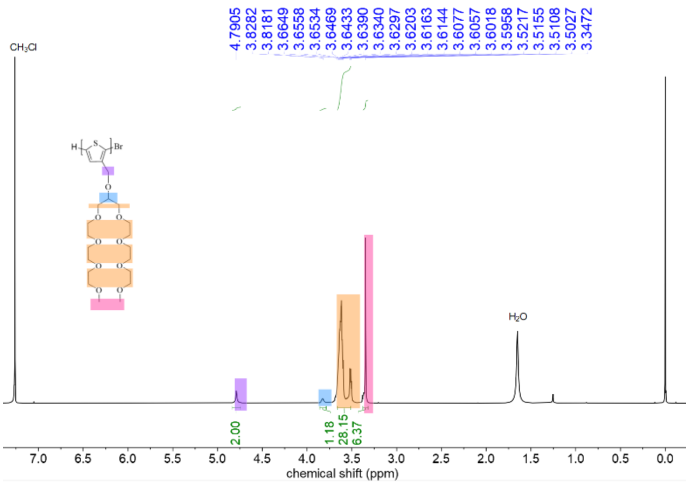

Figure S7. ^{1}H NMR of P3MBTEMT in $CDCl_3$.

2.2.2. Gel Permeation Chromatography (GPC)

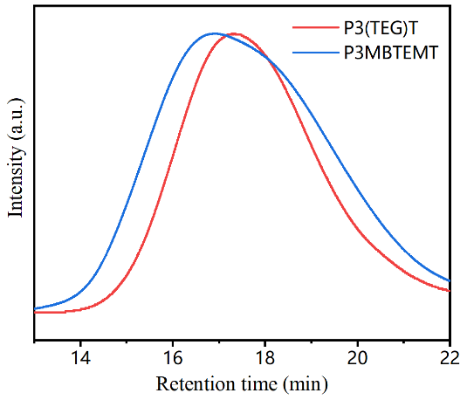

Figure S8. GPC curve of P3(TEG)T and P3MBTEMT.

2.2.3. Thermogravimetric Analysis (TGA)

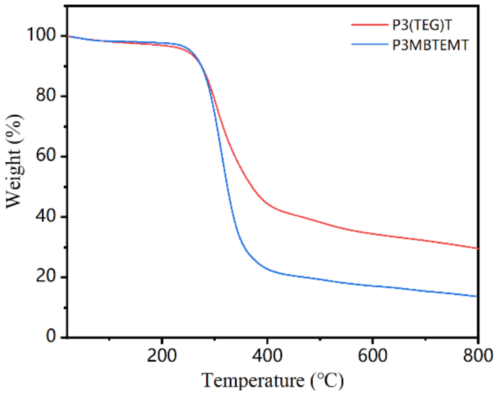

Figure S9. TGA curve of P3(TEG)T and P3MBTEMT.

Table S1. Molecular weights and thermal stabilities of P3(TEG)T and P3MBTEMT.

Polymer	M_n [kDa][a)]	M_w [kDa][b)]	PDI[c)]	$T_{d,5\%}$ [°C][d)]	$\lambda^{sol}_{abs,max}$ [eV][e)]	$\lambda^{sol}_{abs,max}$ [eV][f)]
P3(TEG)T	18.4	28.4	1.53	32	445	431

P3MBTEMT	18.9	34.8	1.84	29	442	447

[a), b), c)]Obtained from the GPC results, M_n: Number-average molecular weight; M_w: weight-average molecular weight, PDI: polydispersity. [d)]Estimated from the TGA curves. [e)]Measured in the THF solution at room temperature. [f)] Measured in the film at room temperature.

2.2.4. Polymer/SWCNTs composite films and devices preparations

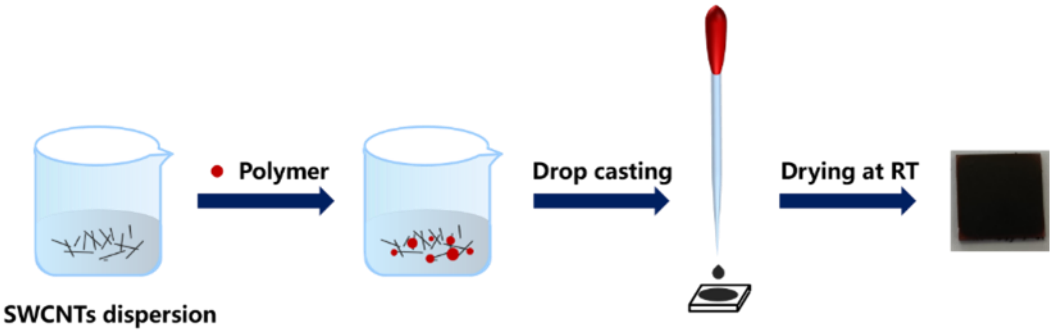

Figure S10. Preparation process of composite film materials.

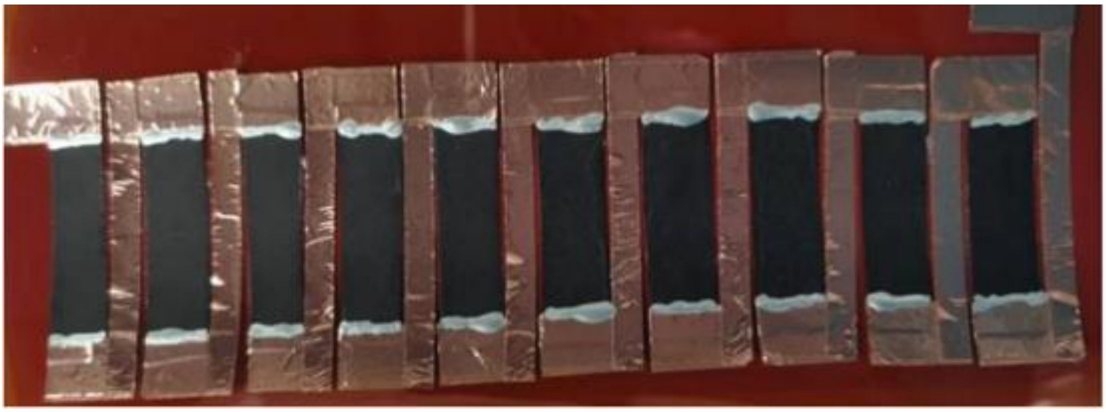

Figure S11. Physical picture of composite film thermoelectric device.

2.2.5. TE properties

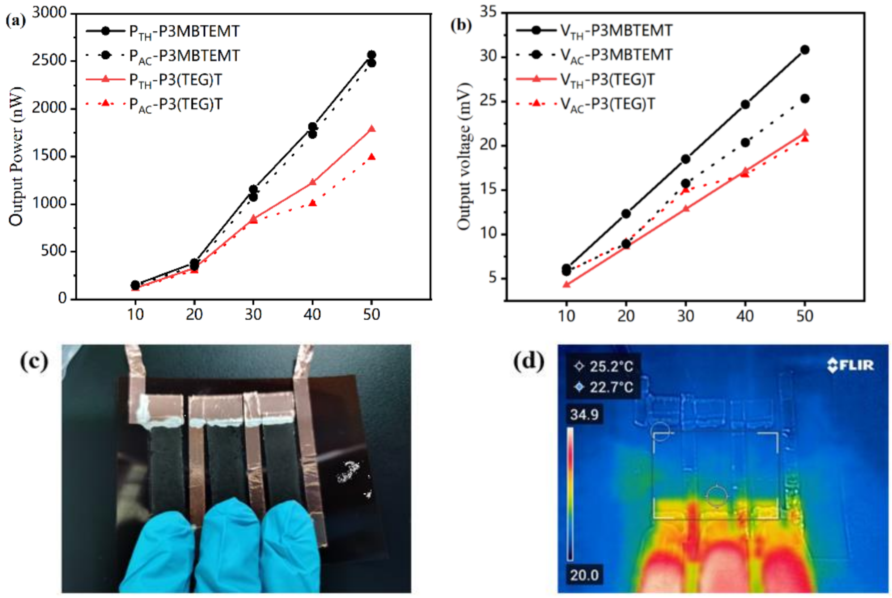

Figure S12. Curve of theoretical open circuit voltage (V_{TH})、actual open circuit voltage (V_{AC})、theoretical output power (P_{TH}) and actual output power (P_{AC}) for a) P3(TEG)T/SWCNTs and b) P3MBTEMT/SWCNTs at different temperature(ΔT). c) Physical picture of simple P3MBTEMT/SWCNTs-0.9 device. (d) infrared imaging of contact device surface.

Table S2. Summary of thermoelectric parameters of reported poly(thiophene)s/SWCNTs composites.

Time	Nanocomposite	Casting method	Post-treatment	*σ* [S cm^{-1}]	*S* [μV K^{-1}]	PF [μW m^{-1} K^{-2}]	Ref.
2013	P3HT /SWCNTs	drop-casting	-	275	32	28	[1]
2013	P3HT /SWCNTs	drop-casting	$FeCl_3$	1130	29	95	[1]
2015	P3HT /SWCNTs	bar-coating	-	501	37.5	71.8	[2]
2015	P3HT /SWCNTs	bar-coating	-	141	56.1	44.4	[3]
2015	P3HT /SWCNTs	bar-coating	immerse $FeCl_3$	638	40	103	[3]
2015	P3HT /SWCNTs	bar-coating	spin $FeCl_3$	2760	31.1	267	[3]
2018	P3HT /SWCNTs	drop-casting	$Fe(TFSI)_3$	579	21.5	49	[4]
2018	rrP3HT /SWCNTs	mold	-	170	45	37	[5]
2018	rrP3HT /SWCNTs	mold	I_2	1722	27	148	[5]

2018	raP3HT /SWCNTs	mold	I_2	85	19	3	[5]
2019	P3HT /SWCNTs	drop-casting	$HClO_4$	118	44.3	23.2	[6]
2019	P3HT /SWCNTs	mold	-	130.8	38.7	19.6	[7]
2019	PTEG /SWCNTs	solvent evaporation	Thermally cleaved	188	38.8	28.8	[8]
2020	P3HT /SWCNTs	drop-casting	-	699.6	43	65	[9]
2020	PMEET /SWCNTs	drop-casting	-	219.1	58	121	[9]
2021	P3HT /SWCNTs	drop-casting	-	506.9	61.2	190.6	[10]
2023	P3HT /SWCNTs	Spin-coating	-	396.8	68.1	172.2	[11]
2023	P3EHT /SWCNTs	Spin-coating	-	352.6	69.5	170.3	[11]
2023	P3EHTT /SWCNTs	Spin-coating	-	486.5	65.2	202.7	[11]
2023	P3HDTT /SWCNTs	Spin-coating	-	745.5	68.0	307.7	[11]
2023	P3HT /SWCNTs	Spray-coating	-	1170	41.8	204	[12]
2023	P3(TEG)T /SWCNTs	drop-casting	-	1368	39.6	215.1	This work
2023	P3MBTEMT /SWCNTs	drop-casting		1312	58.2	447.0	This work

(**Table S2** supplements the summary made by Lin [11].).

References

1. C. Bounioux, P. Díaz-Chao, M. Campoy-Quiles, M.S. Martín-González, A.R. Goñi, R. Yerushalmi-Rozen, C. Müller, Thermoelectric composites of poly(3-hexylthiophene) and carbon nanotubes with a large power factor, Energy Environ. Sci. 6 (2013) 918–925. https: //doi.org/10.1039/C2EE23406H.
2. W. Lee, C.T. Hong, O.H. Kwon, Y. Yoo, Y.H. Kang, J.Y. Lee, S.Y. Cho, K.-S. Jang, Enhanced Thermoelectric Performance of Bar-Coated SWCNT/P3HT Thin Films, ACS Appl. Mater. Interfaces. 7 (2015) 6550–6556. https://doi.org/10.1021/acsami.5b00626.
3. C.T. Hong, W. Lee, Y.H. Kang, Y. Yoo, J. Ryu, S.Y. Cho, K.-S. Jang, Effective doping by spin-coating and enhanced thermoelectric power factors in SWCNT/P3HT hybrid films, J. Mater. Chem. A. 3 (2015) 12314–12319. https://doi.org/10.1039/C5TA02443A.
4. S. Qu, M. Wang, Y. Chen, Q. Yao, L. Chen, Enhanced thermoelectric performance of CNT/P3HT composites with low CNT content, RSC Adv. 8 (2018) 33855–33863. https: //doi.org/10.1039/C8RA07297C.
5. M. Tonga, L. Wei, E. Wilusz, L. Korugic-Karasz, F.E. Karasz, P.M. Lahti, Solution-fabrication dependent thermoelectric behavior of iodine-doped regioregular and regiorandom P3HT/carbon nanotube composites, Synthetic Metals. 239 (2018) 51–58. https://doi.org/10. 1016/j.synthmet.2018.03.007.

6. X. Li, Z. Zhu, T. Wang, J. Xu, C. Liu, Q. Jiang, F. Jiang, P. Liu, Improved thermoelectric performance of P3HT/SWCNTs composite films by HClO4 post-treatment, Composites Communications. 12 (2019) 128–132. https://doi.org/10.1016/j.coco.2019.01.009.
7. Y.H. Kang, U.-H. Lee, I.H. Jung, S.C. Yoon, S.Y. Cho, Enhanced Thermoelectric Performance of Conjugated Polymer/CNT Nanocomposites by Modulating the Potential Barrier Difference between Conjugated Polymer and CNT, ACS Appl. Electron. Mater. 1 (2019) 1282–1289. https://doi.org/10.1021/acsaelm.9b00224.
8. P. He, S. Shimano, K. Salikolimi, T. Isoshima, Y. Kakefuda, T. Mori, Y. Taguchi, Y. Ito, M. Kawamoto, Noncovalent Modification of Single-Walled Carbon Nanotubes Using Thermally Cleavable Polythiophenes for Solution-Processed Thermoelectric Films, ACS Appl. Mater. Interfaces. 11 (2019) 4211–4218. https://doi.org/10.1021/acsami.8b14820.
9. L. Hao, J. Kang, J. Shi, J. Xu, J. Cao, L. Wang, Y. Liu, C. Pan, Enhanced thermoelectric performance of poly(3-substituted thiophene)/single-walled carbon nanotube composites via polar side chain modification, Composites Sci. Technol. 199 (2020) 108359. https://doi. org/10.1016/j.compscitech.2020.108359.
10. C. Liu, X. Yin, Z. Chen, C. Gao, L. Wang, Improving the thermoelectric performance of solution-processed polymer nanocomposites by introducing platinum acetylides with tailored intermolecular interactions, Chem. Eng. J. 419 (2021) 129624. https://doi.org/10.1016/j. cej.2021.129624.
11. P.-S. Lin, S. Inagaki, J.-H. Liu, M.-C. Chen, T. Higashihara, C.-L. Liu, The role of branched alkylthio side chain on dispersion and thermoelectric properties of regioregular polythiophene/carbon nanotubes nanocomposites, Chem. Eng. J. 458 (2023) 141366. https: //doi.org/10.1016/j.cej.2023.141366.
12. [S.-H. Hong, T.-C. Lee, C.-L. Liu, All-Solution-Processed Polythiophene/Carbon Nanotube Nanocomposites Integrated on Biocompatible Silk Fibroin Substrates for Wearable Thermoelectric Generators, ACS Appl. Energy Mater. 6 (2023) 2602–2610. https://doi. org/10.1021/acsaem.2c04160.

Disclaimer/Publisher's Note: The statements, opinions and data contained in all publications are solely those of the individual author(s) and contributor(s) and not of MDPI and/or the editor(s). MDPI and/or the editor(s) disclaim responsibility for any injury to people or property resulting from any ideas, methods, instructions or products referred to in the content.
